# Visualising and semi-quantitatively measuring brain fluid pathways, including meningeal lymphatics, in humans using widely available MRI techniques

**DOI:** 10.1177/0271678X231179555

**Published:** 2023-05-31

**Authors:** Stefan Sennfält, Michael J Thrippleton, Michael Stringer, Carmen Arteaga Reyes, Francesca Chappell, Fergus Doubal, Daniela J Garcia, Junfang Zhang, Yajun Cheng, Joanna Wardlaw

**Affiliations:** 1 Department of Neurology, Karolinska University Hospital, Stockholm, Sweden; 2 Department of Clinical Neuroscience, Karolinska Institutet, Stockholm, Sweden; 3 Centre for Clinical Brain Sciences, University of Edinburgh, Edinburgh, UK; 4 Department of Neurology, Shanghai General Hospital and Shanghai Jiao Tong University School of Medicine, Shanghai, China; 5 Department of Neurology, West China Hospital and Sichuan University, Chengdu, China; 6 UK Dementia Research Institute at the University of Edinburgh, Edinburgh, UK

**Keywords:** Meningeal lymphatics, brain fluid drainage, MRI, glymphatics, small vessel disease

## Abstract

Brain fluid dynamics remains poorly understood with central issues unresolved. In this study, we first review the literature regarding points of controversy, then pilot study if conventional MRI techniques can assess brain fluid outflow pathways and explore potential associations with small vessel disease (SVD). We assessed 19 subjects participating in the Mild Stroke Study 3 who had FLAIR imaging before and 20–30 minutes after intravenous Gadolinium (Gd)-based contrast. Signal intensity (SI) change was assessed semi-quantitatively by placing regions of interest, and qualitatively by a visual scoring system, along dorsal and basal fluid outflow routes. Following i.v. Gd, SI increased substantially along the anterior, middle, and posterior superior sagittal sinus (SSS) (82%, 104%, and 119%, respectively), at basal areas (cribriform plate, 67%; jugular foramina, 72%), and in narrow channels surrounding superficial cortical veins separated from surrounding cerebrospinal fluid (CSF) (96%) (all p < 0.001). The SI increase was associated with higher intraparenchymal perivascular spaces (PVS) scores (Std. Beta 0.71, p = 0.01). Our findings suggests that interstitial fluid drainage is visible on conventional MRI and drains from brain parenchyma via cortical perivenous spaces to dural meningeal lymphatics along the SSS remaining separate from the CSF. An association with parenchymal PVS requires further research, now feasible in humans.

## Introduction

The volume of cerebrospinal fluid (CSF) in humans is often approximated to around 150 ml, with 500 ml produced every day, primarily by the choroid plexuses in the cerebral ventricles.^1^ The CSF circulates through the central nervous system (CNS) and eventually drains back into the systemic circulation. However, the fluid pathways and the mechanism for its drainage are unclear. The ‘traditional model’ suggests that CSF exits the ventricles via foramina of Magendie and Lushka, then circulates over the brain’s outer surface primarily providing buoyancy and protection before being reabsorbed into venous blood at the major venous sinuses (primarily the superior sagittal sinus [SSS]) through intravenous projections called arachnoid granulations.^2^ In this model, little attention is paid to the interaction between CSF and brain parenchyma, but the latter has received increased attention in recent years, leading to the emergence of the ‘glymphatic’ hypothesis. This proposes a large volume inflow of CSF via periarteriolar spaces into the brain parenchyma and thence through the interstitial space, with outflow from the parenchyma via perivenous spaces, carrying with it solutes and waste.^3^ Also, the idea of the arachnoid granulations being the main CSF outflow route from the CNS has been challenged in light of little experimental support.^1^ Meanwhile, there is mounting evidence for the importance of other outflow routes, such as via lymphatic channels accompanying some cranial/spinal nerves, major vessels, or running along other meningeal surfaces to exit the cranium.^1^

There is now a substantial body of literature on brain fluid drainage, but the bulk of evidence comes from animal experiments, results are inconclusive and often contradicting. Central issues remain unresolved, such as whether the interstitial fluid exiting the brain parenchyma mixes with the ‘clean’ CSF. In recent years, more studies have tried to use magnetic resonance imaging (MRI) to visualise brain fluid dynamics in humans in vivo.^4–20^ However, methods are varied, often complex and thus not widely available, and systematic quantitative measurement is often lacking or is poorly developed. CSF volume, perivascular spaces (PVS), blood-brain barrier (BBB) leakage, etc, can now be assessed visually or computationally with reasonable reliability, increasing the need for reliable methods to assess the number, size, signal changes, etc, of the efflux side of the brain fluid drainage system in research. While several recent studies report measurements of individual components of the meningeal lymphatic and CSF drainage systems, none described a comprehensive and practical assessment method.

This study aims first to review the literature focusing on the specific points of controversy on brain fluid outflow pathways and secondly to investigate methods of using conventional and widely applied MRI techniques to explore these pathways and provide a comprehensive assessment. Thus, in an effort to determine which fluid drainage pathways might be most important for efficient brain fluid management in humans, and to develop a robust clinically applicable measurement method, we performed brain MRI using the relative differences in fluid signal on T2 fluid-attenuated inversion recovery (FLAIR) between pre- and post-gadolinium (Gd) images to identify the potential fluid pathways. We also devised and tested practical methods to assess the fluid pathways for use in larger studies in volunteers and patients with disorders potentially related to impaired fluid drainage. Finally, we performed exploratory analyses to test for potential relations between signal intensity (SI) or its change and markers of small vessel disease (SVD), such as PVS and white matter hyperintensities (WMH), both associated with impaired CSF pulsatility,^21^ which may be important for driving interstitial fluid clearance.^22^

## Subjects and methods

### Review

We searched PubMed for original studies, review papers and the authors’ reference libraries to identify papers describing assessment of fluid inflow to and outflow from the brain and cranial compartment in humans as well as imaging methods (see supplementary table 1 for initial search terms and dates). We extracted information on subjects, method of imaging, quantification of the interstitial, meningeal or other brain and cranial outflow pathways. We discuss main summary findings in turn.

### Subjects

We used data from 19 subjects participating in the Mild Stroke Study 3 (MSS-3),^23^ a prospective observational cohort study of SVD in adults presenting with lacunar or mild cortical ischaemic stroke. The MSS-3 aims to assess cerebrovascular dysfunctions in SVD (BBB, cerebrovascular reactivity, vascular and CSF pulsatility), long term SVD lesion progression and clinical and cognitive outcomes. The protocol is published,^23^ the study has ethics (REC 18/SS/0044, IRAS ID 235737) and R&D (2018/0084) approvals and all patients gave written informed consent.

### Imaging

In the MSS-3, at one to three months post-stroke, subjects undergo contrast-enhanced MRI to assess BBB leakage using intravenous (IV) Gadolinium (Gd) and other markers of cerebral vascular status and function. All patients underwent standard clinical, cognitive, physiological, and MRI assessments as described.^23^ In a subset of subjects, we added several post-contrast images at the end of the BBB imaging run to visualise meningeal lymphatics and related structures. The results from these subjects are the focus of the present paper.

All patients were imaged on the same 3 T MRI scanner (MAGNETOM Prisma, Siemens Healthcare, Erlangen) which underwent a continuous quality control monitoring programme. Though several MRI sequences were acquired in the MSS-3 study, here we only present those relevant to this paper. Imaging was performed as detailed before and 20–30 minutes after administration of 0.1 mmol/kg body weight of Gd based contrast agent Gadobutrol (except in one subject where this time was 46 minutes). 2 D coronal T2-FLAIR images were obtained using a motion-compensated PROPELLER fast spin-echo sequence with fat saturation and a saturation band placed inferiorly to the imaging volume (TR/TE/TI = 8000/129/2370 ms, 36 × 4-mm slices, 1.2 mm slice gap, 0.6 mm in-plane resolution, parallel imaging acceleration factor 2, acquisition time 4 m18 s). 2 D axial T2-FLAIR images were obtained using the same technique (TR/TE/TI = 6600/129/2140 ms, 30 × 4-mm slices, 1.2 mm slice gap, 0.7 mm in-plane resolution, parallel imaging acceleration factor 2, acquisition time 3 m 33 s).

### Measurement of signal intensity

All measurements were performed by a clinical neurology resident (SS), who was blind to all other clinical and imaging data, using Philips Carestream DICOM viewing software (© 2022 Koninklijke Philips N.V., version 12.2.5.00397), a typical and widely used clinical radiological viewing platform. Measurements were supervised by a senior neuroradiologist (JW) with many years of experience in diagnostic neuroradiology who was also blinded to all other data.

We assessed structures (1) semi-quantitatively by manually placing regions of interest (ROIs), measuring the SI in arbitrary units, and (2) qualitatively by visually rating the SI on a scale of 0–4 (only integrals) guided by standardised reference images (Supplementary table 2). All measurements were conducted pre-and post-contrast. Although FLAIR signal intensity is not quantitative per se, since we reported and compared SI before and after contrast within each patient acting as their own control, the measurement was considered semi-quantitative. All measurements were repeated three times, six weeks apart, and the average was taken. Reliability was assessed (see statistics section).

We chose several areas of assessment for which there is evidence indicating these as potential sites of fluid outflow and meningeal lymphatic location (see examples in Table 1):^1,4,16^ basal outflow routes (the cribriform plate and the jugular foramina), dorsal outflow routes (at three sites along the SSS [anterior, middle, and posterior] and at the confluence of major dural venous sinuses, the Torcula). Lymphatic vessels were identified in cross section as punctate hyperintensities on pre- and post-contrast T2 FLAIR and assessed at predetermined sections defined by anatomical landmarks: in the coronal plane for the anterior/middle SSS and the cribriform plate, and in the axial plane for the posterior SSS and the jugular foramina. In most instances, except for the jugular foramina, two or three punctate hyperintensities could be identified. The SI at the areas of assessment was measured by placing a ROI of approximately 1 mm^3^ at each of these and extracting the average (SI) values. For the jugular foramina (where the hyperintensity was more diffusely distributed) a larger ROI of approximately 7 mm^3^ was placed over the pars nervosa at either side (right and left) and the average was taken.

**Table 1.** Details of the measurement of brain fluid outflow.

	Anatomical landmark	Projection	Plane of measurement	Measure	Example images
Superior Sagittal Sinus					
*Anterior*	Anterior temporal pole	Coronal	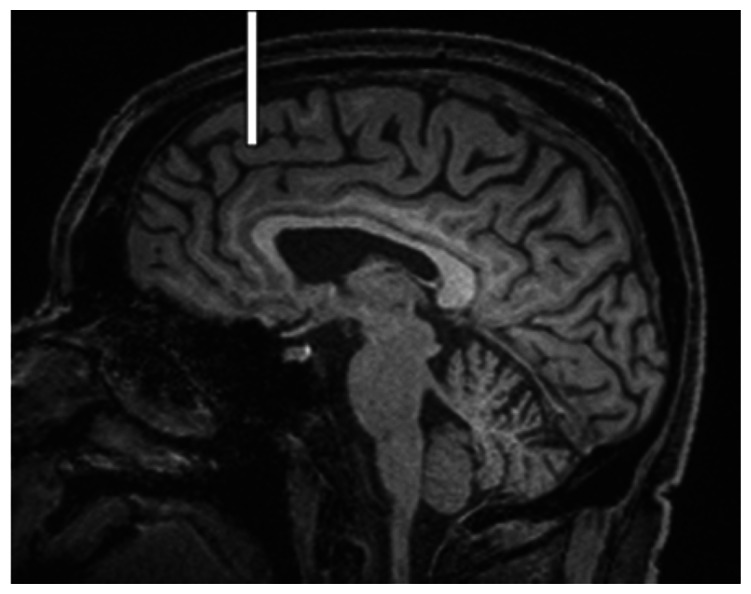	Pre- and post-contrast SI of meningeal lymphatics in cross section. Semi-quantitatively by placing 1 mm^3^ ROIs on 2 or 3 punctate hyperintensities. Qualitatively by visual scoring (0–4).	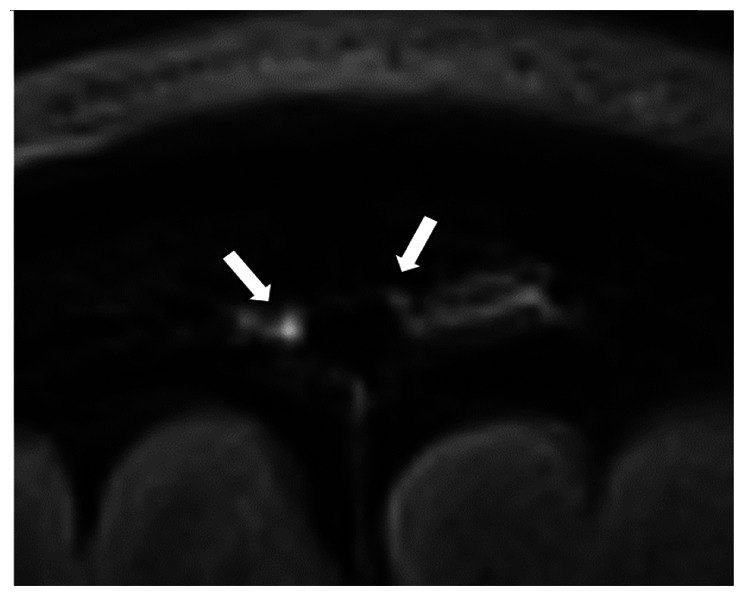
*Middle*	4^th^ ventricle	Coronal	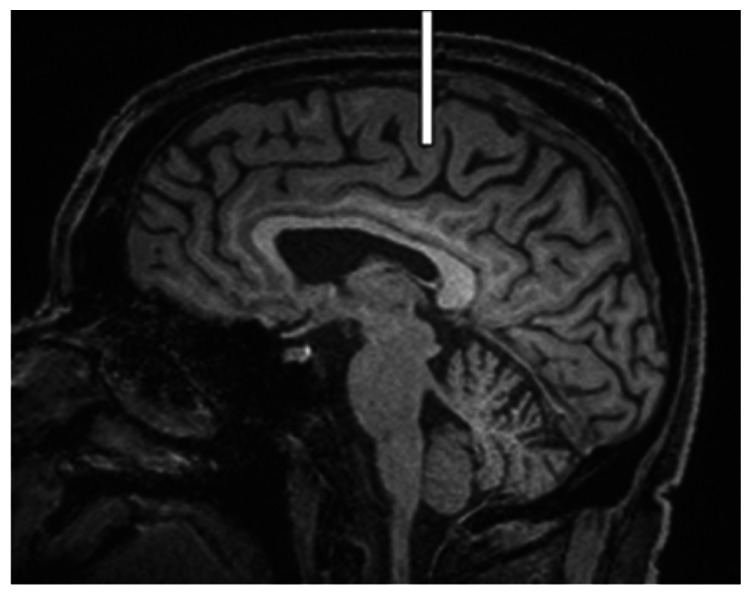	Pre- and post-contrast SI of meningeal lymphatics in cross section. Semi-quantitatively by placing 1 mm^3^ ROIs on 2 or 3 punctate hyperintensities. Qualitatively by visual scoring (0–4).	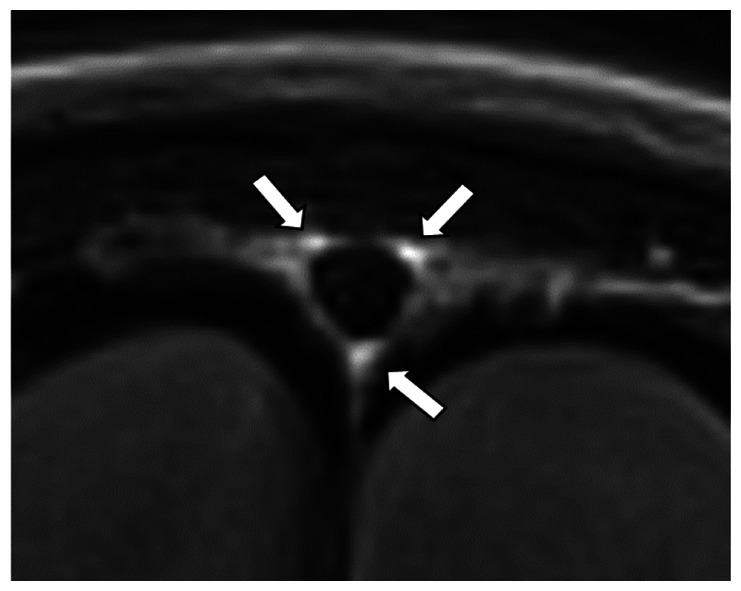
*Posterior*	Floor of lateral ventricles	Axial	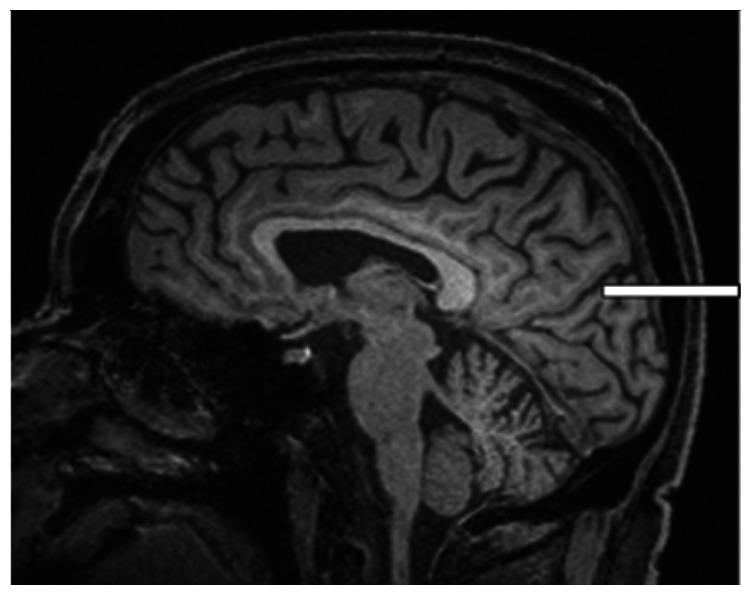	Pre- and post-contrast SI of meningeal lymphatics in cross section. Semi-quantitatively by placing 1 mm^3^ ROIs on 2 or 3 punctate hyperintensities. Qualitatively by visual scoring (0–4).	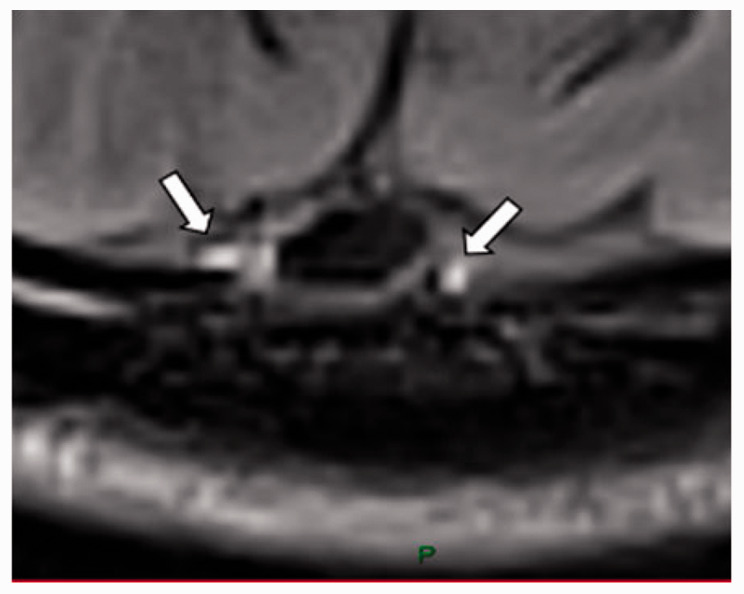
Torcula	Confluence of the superior sagittal sinus and the straight sinus, anterior to the transverse sinus.	Coronal	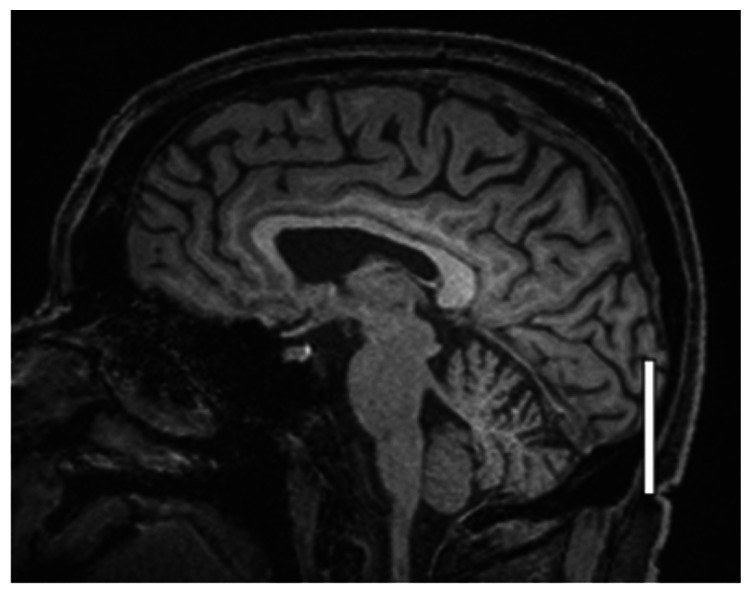	Pre- and post-contrast SI of meningeal lymphatics in cross section. Semi-quantitatively by placing 1 mm^3^ ROIs on 2 or 3 punctate hyperintensities. Qualitatively by visual scoring (0–4).	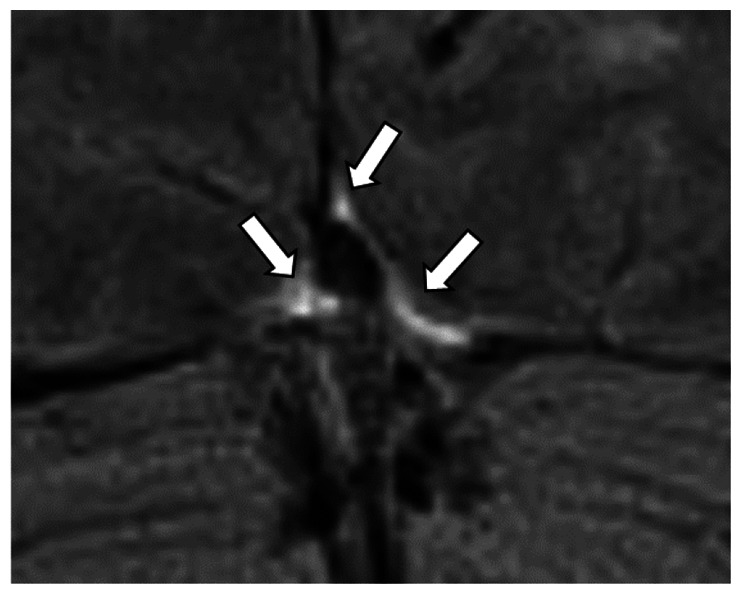
Cribriform plate		Coronal	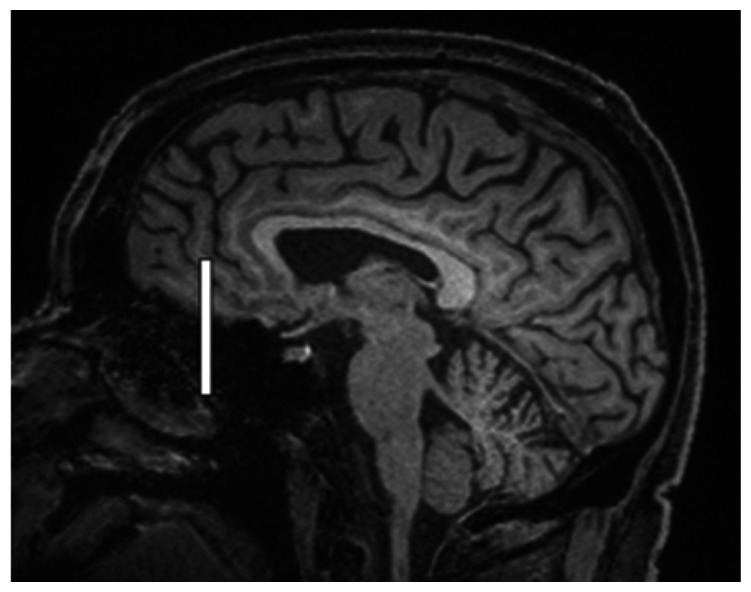	Pre- and post-contrast SI of meningeal lymphatics in cross section. Semi-quantitatively by placing 1 mm^3^ ROIs on 2 punctate hyperintensities. Qualitatively by visual scoring (0–4).	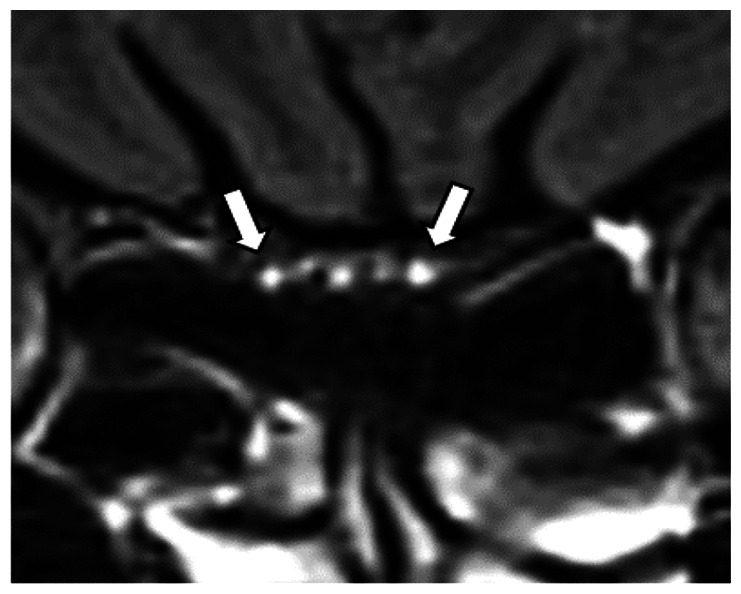
Jugular foramina		Axial	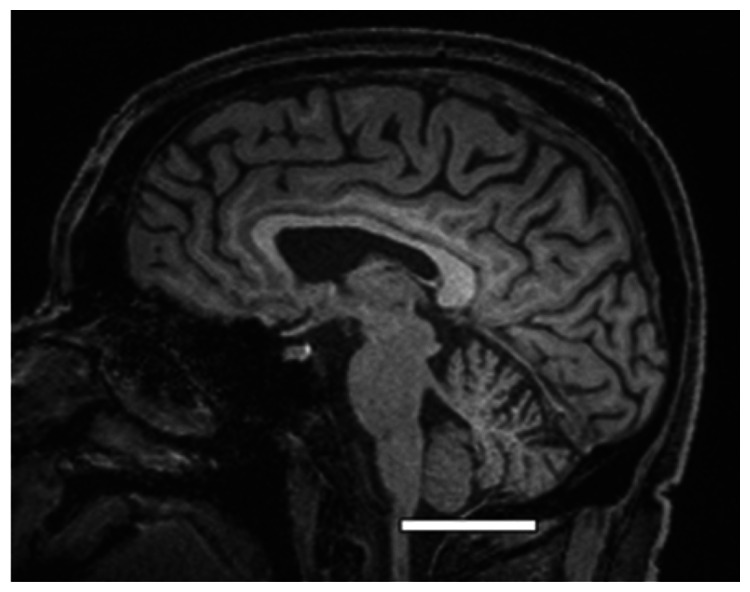	Pre- and post-contrast SI of the pars nervosa of the jugular foramen. Semi-quantitatively by placing 7 mm^3^ ROIs. Qualitatively by visual scoring (0–4).	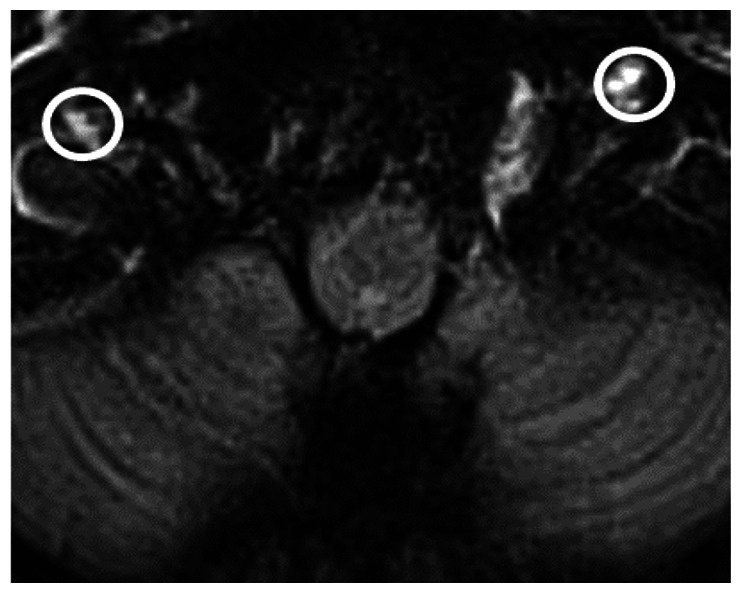
Superficial cortical perivenous spaces	–	Coronal	–	Pre- and post-contrast SI of perivenous hyperintensities in longitudinal section. Semi-quantitatively by placing two pairs of 1 mm^3^ ROIs at either side of the vessel. Qualitatively by visual scoring (0–4).	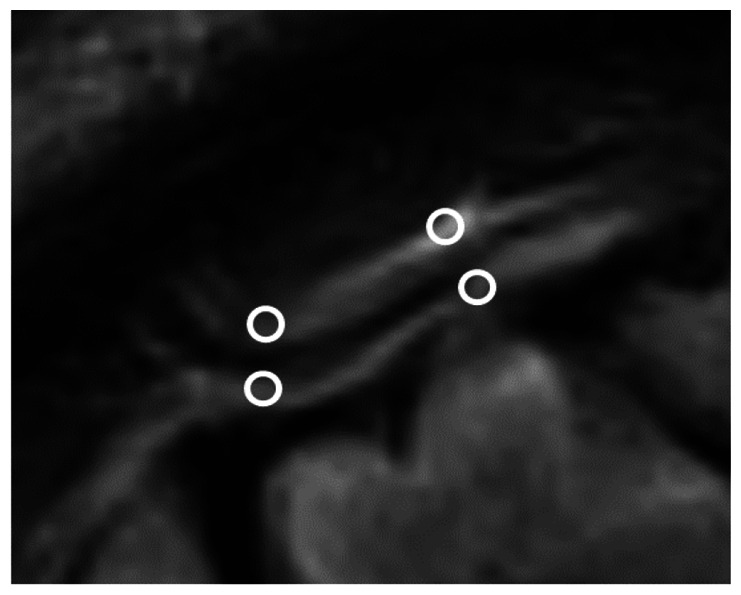
’Negative’ reference (NAWM)	Anterior temporal pole. Upper corona radiata.	Coronal	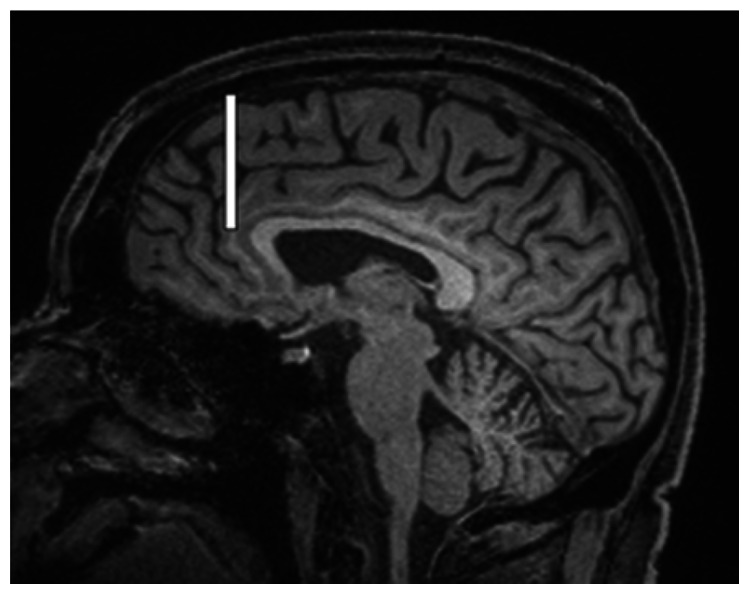	Pre- and post-contrast SI. Semi-quantitatively by placing a 5 mm^3^ ROI at either side of the midline.	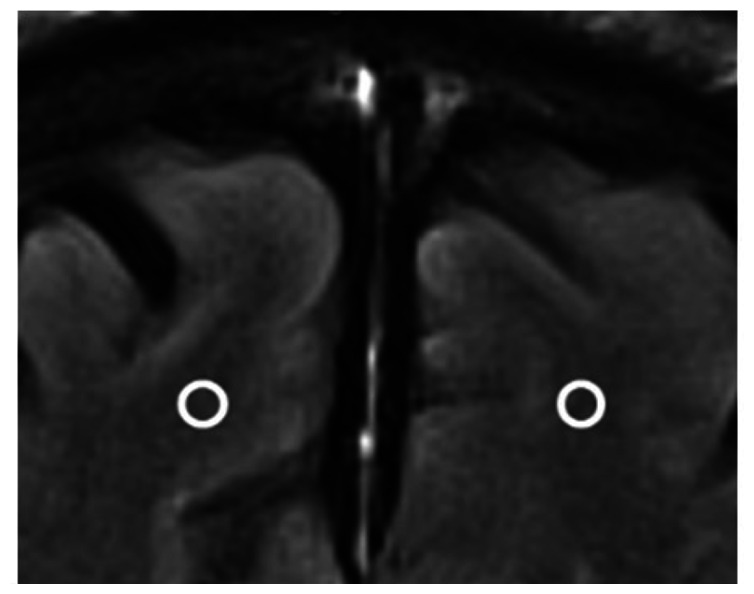
‘Positive’ reference (Pituitary stalk)	–	Axial	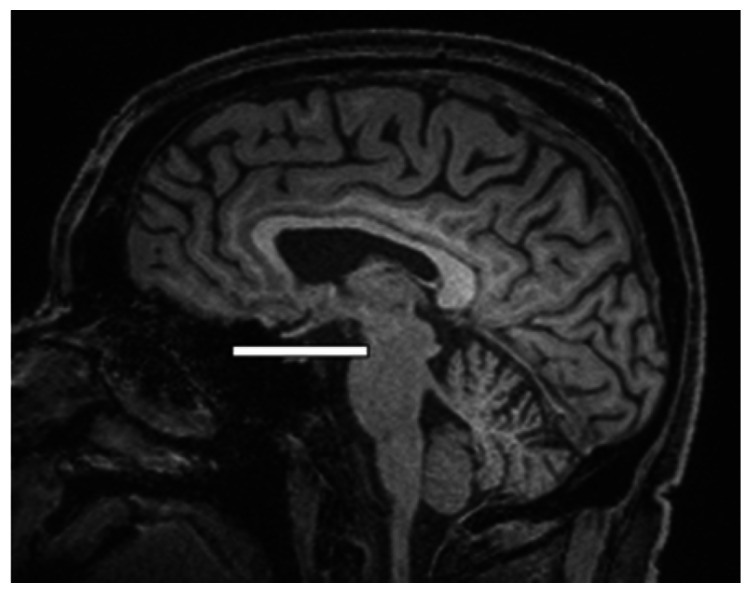	Pre-and post-contrast SI. Semi-quantitatively by placing a 2 mm^3^ ROI.	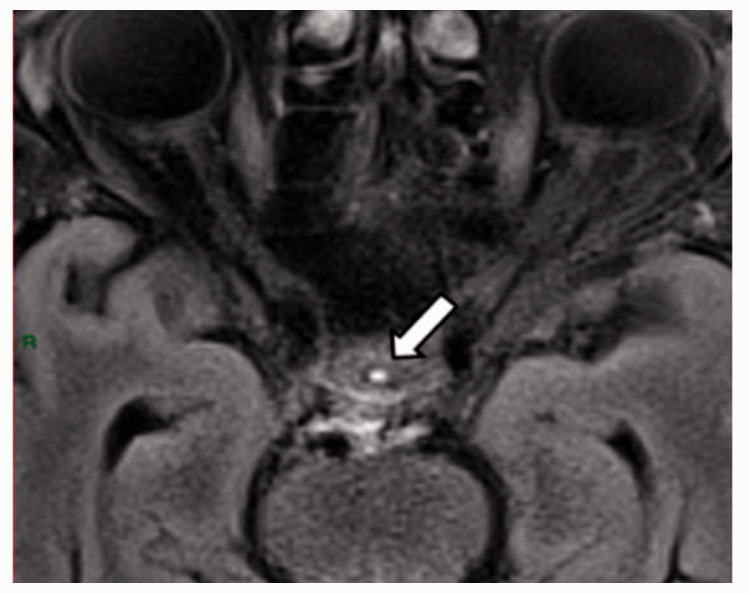

The white bar in the column ‘plane of measurement’ indicates location of scan slice, in the orientation indicated in the column ‘projection’ and shown in the column ‘example images’. In the ‘example images’ the arrows indicate the meningeal lymphatics seen as punctate, linear, or sheet hyperintensities on T2 FLAIR post-contrast that were measured by placing ROIs. For the jugular foramina, superficial cortical perivenous spaces, and normal appearing white matter, ROIs are indicated by circles.

FLAIR: fluid-attenuated inversion recovery; NAWM: normal appearing white matter; ROI: region of interests; SI: signal intensity.

We also assessed the SI of superficial cortical perivenous spaces on T2 FLAIR which appeared as linear hyperintensities (‘tramlines’) running along (parallel with) the veins as they pass over the cortical surface towards the venous sinuses (Table 1). We only included perivenous hyperintensities with a traceable length of ≥2 cm on both sides of the vessel on post-contrast imaging and identifiable anatomically on pre-contrast imaging. Although clearly visible in the axial plane, we found that longer vessel lengths were more often visible on coronal imaging. Therefore, in the present work, we measured perivenous space SI in the coronal plane, often over several sections. We measured two pairs (at least 1 cm apart) of approximately 1 mm^3^ ROIs at either side of the vessel and calculated the average. Since the vessel was often tracked over several sections the pairs of ROIs were not always found in the same section.

We also measured two reference points to provide a minimum and maximum signal change between the pre- and post-contrast images (examples, see Table 1): normal appearing white matter (NAWM), as a ‘negative’ control, since little, if any, visible contrast enhancement is expected in normal subjects, and the pituitary stalk as a ‘positive’ control since significant contrast enhancement is expected in normal subjects.

Finally, several other areas previously reported as putative outflow sites were assessed but discontinued due to difficulty in achieving consistent and systematic visualisation and measurement due to anatomical variation. These areas were the sigmoid and transverse sinuses, the optic nerves, cranial nerves VII/VIII, the cavernous sinus, and the trigeminal cave.

### Other demographic and imaging variables

Patient demographic, stroke related, and other variables were collected as described in the published protocol.^23^ All baseline imaging was assessed by trained researchers who were masked to clinical, demographic and cognitive data, using established visual and computational methods, and who were not involved in the measurement of the fluid drainage pathways, as described in the protocol.^23^ They assessed features of SVD including WMH using the Fazekas score, and PVS in the basal ganglia and centrum semiovale regions using a validated visual score, lacunes, microbleeds and brain volume loss, according to STandards for ReportIng Vascular Changes on NEuroimaging (STRIVE) criteria.^24^

### Statistical methods

All 19 subjects were included for analysis of all variables other than for superficial cortical perivenous spaces where image quality limited analysis to 16 patients.

Categorical data were summarised as proportions (percent) and continuous variables were reported as median with the interquartile range (IQR, defined as the 25th–75th percentile). Throughout the paper, decimals were rounded to two.

Both qualitative and semi-quantitative measurement of SI was performed three times at least six weeks apart. The choice of methodology for assessing inter/intrarater reliability when there are more than two sets of measurements is debated. Therefore, we used several commonly used methods as described below.

For semi-quantitative measurement (continuous data) we used the Limits of agreement (Bland-Altman) method,^25^ analysing each pair of scorings separately: the first and second, the first and third, and the second and third, yielding the mean difference (95% confidence interval [CI]), including assessment of significant deviation from zero using the one-sample t-test. We also performed linear regression analyses for each pair of values to test if the discrepancy between values within pairs varied through the range of measurements. The mean value of each pair was used as the independent variable while the difference was used as the dependent variable. In addition, to produce a single value indicating reliability of the semi-quantitative scorings we calculated the intraclass correlation coefficient (ICC) (two-way mixed model), testing for absolute agreement. Values greater than 0.75 were considered good reliability.^26^ To assess reliability of the measurements for the qualitative SI scoring (ordinal scale) the Fleiss’ kappa method was used. Values between 0.4 and 0.6, and greater than 0.6 were considered moderate and good reliability, respectively.^27^ However, these proposed cut-offs are debated and should only be viewed as a rough indication.

For semi-quantitative and qualitative assessment of SI the average from the three measurements was taken for each individual and was then reported at group level as median and IQR (25–75th percentiles), except in supplementary table 5 where the qualitative scores were shown as mean (95% Cl) since differences between measurements on the 0–4 scale would be difficult to appreciate if reported as median. Both the absolute and the relative (the proportion of pre-contrast SI) change in SI between pre-and post-contrast imaging were reported. The change in SI was first calculated for each individual (average of the three measurements) and then reported at group level as medians and the paired samples t-test (two-tailed) was used to test for significance.

Correlation between semi-quantitative SI values in the different areas of assessment was explored using the Pearson’s correlation coefficient.

Linear regression analysis was used to assess association 1) between semi-quantitative SI values and qualitative SI scoring, 2) between the time from administration of contrast to imaging and the relative change in SI, and 3) between change in SI (the average from all seven areas of assessment) and patient characteristics (sex and age) as well as markers of SVD. We constructed separate models for the total number of visible PVS in the basal ganglia and centrum semiovale, the summed Fazekas score, and a total SVD score (a summed assessment of several features on MRI such as presence of lacunes, WMH, cerebral microbleeds, and PVS as described previously^28^). All three models included one of these as the independent variable, age and sex as covariates, and the dependent variable was the average (merged) relative change in SI for all areas of assessment. A separate model was constructed including only sex and age to specifically explore the effect of these two variables. Since the sample size was small (n = 19) the number of predictors was limited to a maximum of three. For all linear regression analyses, the standardized Beta coefficient (Std. Beta) was reported.

All statistical analyses were conducted in IBM SPSS Statistics version 28.

## Results

### Review

#### Fluid inflow to brain parenchyma

**Figure 1.**
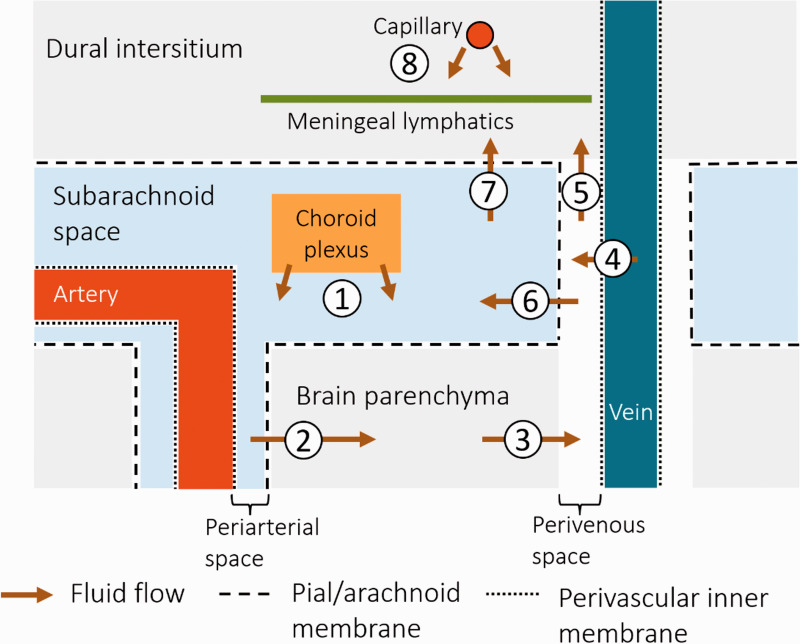
Schematic overview of potential brain fluid drainage pathways. The nature of these is not fully elucidated and this diagram illustrates some potentially important proposed routes. (1) Cerebrospinal fluid (CSF) is formed by choroid plexi and circulates in the subarachnoid space. (2) The CSF enters the brain parenchyma via perivascular spaces (likely primarily arteriolar), and becomes interstitial fluid that then (3) exits via perivascular spaces (perhaps primarily perivenous). (4) In addition, fluid leakage from vein vessel walls into the surrounding perivenous spaces has been suggested. The route out of the cranium is debated: the interstitial fluid might be drained by a separate system, e.g., (5) to the dural intersitium via perivenous spaces where it enters meningeal lymphatic vessels, or (6) it might mix with the CSF in the subarachnoid spaces, thus mixing the ’dirty’ effluent with the ’clean’ CSF, and then (7) possibly entering the dural interstitium or meningeal lymphatics, or exiting the cranium by other routes (e.g., perineural spaces). (8) Also, some fluid might be exiting via the dural interstitial capillaries and entering the dural meningeal lymphatics without interaction with the CSF.

In the ‘glymphatics’ proposal, fluid is suggested to flow in a continuous space from the main site of formation by choroid plexi in the ventricles, out of the ventricles to the extracerebral subarachnoid spaces, thence into (primarily periarteriolar) PVS in the brain parenchyma (Figure 1). Aided by arterial pulsations and Aquaporin 4 channels,^22,29^ the fluid is then thought to move into the brain interstitial extracellular space where it can mix with any fluid that has leaked from the microvasculature or has formed as a by-product of cellular metabolism. The PVS are central to this model, in which they function as conduits for (periarteriolar) inflow and (perivenular) outflow of fluid from the brain parenchyma.^3^ As vessels travel deep into cerebral tissue, they are surrounded by a PVS encased by a leptomeningeal sheath.^30^ That these spaces are in communication with the subarachnoid space is suggested by multiple experiments in animals and a few in humans where tracers administered intrathecally appear to move along penetrating arterioles.^20,30–32^ However, the connectivity of the PVS to outflow paths and the anatomy of the surrounding meningeal envelope is less clear, with possible variation between arteries versus veins and between different parts of the brain.^30,33,34^

#### Fluid efflux from the brain parenchyma

Parenchymal interstitial fluid eventually drains to the venous circulation and/or CNS-associated lymphatics, but the anatomical pathways from the parenchyma are debated. The most direct and specific way of assessing these outflow pathways is to track the distribution of a tracer injected into the brain parenchyma. This is generally not feasible in living humans, and is also not physiological, but has frequently been performed in rodents and suggested a system of parenchymal outflow pathways now referred to as intramural periarterial drainage (IPAD) associated with blood vessels.^35–37^ Of note, the IPAD pathway suggests that interstitial molecular effluent moves along arteriolar basement membranes towards the brain surface, i.e., in the opposite direction to the proposed influx of CSF into PVS around the perforating arterioles in the glymphatic hypothesis.

Many models of brain fluid flow imply that fluid flowing out from the parenchyma mixes with the CSF pool overlying the brain,^1,3,37,38^ and hence do not distinguish if clean fluid inflow and dirty fluid outflow systems are separated. Thus, it is currently an open question whether interstitial fluid: (1) mixes with the common CSF pool and then drains out of the cranium, (2) drains via a dedicated outflow system separate from ‘clean’ CSF; or (3) a mix of these options (Figure 1). If so, is one system more quantitatively important, and do they serve different functions?

Intuitively, it would seem to be mechanistically favourable for outflow to occur via a low-pulsatile low resistance (i.e., venous) system as opposed to the higher-pulsatility periarteriolar spaces which could instead work to push fluid towards the parenchyma. Also, different outflow routes would keep the main CSF pool that bathes the brain surfaces clean, rather than potentially bathing the brain in ‘contaminated’ parenchymal outflow. However, although there are indeed studies demonstrating passage of interstitial fluid, hydrophilic and lipophilic molecules out of the brain via perivascular spaces in rodents,^31,37,39^ it has not been determined whether these outflow routes are predominantly periarteriolar or perivenous.

In support of a parenchymal drainage remaining separate from the CSF are animal experiments quantifying outflow from the parenchyma to the CSF. In several studies using different size tracers administered into the rodent caudate nucleus, only a small portion of the tracer that cleared from the brain could be recovered from CSF,^36,40,41^ suggesting alternative pathways of outflow that avoid CSF contamination. Also, in opportunistic human experiments, tracer administered intrathecally into the spinal CSF has been demonstrated to accumulate centripetally in the brain interstitium many hours after intrathecal injection, during which time the signal in CSF declines continuously (without a second delayed peak to indicate recirculation, which speaks against a significant efflux to CSF from the parenchyma.^20,42^

Importantly, a separation of influx and efflux would require a barrier separating outflow routes (e.g., via perivenous spaces), from the CSF. There are few studies exploring this, but there are indications that while some penetrating arterioles (primarily cortical) are invested by two membrane layers, intra-parenchymal venules are only surrounded by a single membrane.^33^ This is interesting since a double layered meningeal sheath (including an invagination of the pial membrane) might form a space surrounding the artery on all sides in direct communication with the subarachnoid CSF spaces (Figure 1). Conversely, the perivenous space would instead be continuous with the subpial space and separated from the subarachnoid CSF by the pial membrane. However, whether this separation continues as the veins traverse the subarachnoid spaces en route to the dural sinuses has received little recent attention.

#### Fluid efflux from the cranium

The arachnoid granulations were long thought to be essential for drainage of CSF, which is now generally accepted not to be the case.^1^ For instance, there is wide variation of number and size of arachnoid granulations without apparent consequences for CSF physiology.^43^ Nevertheless, tracer administered into the brain parenchyma can be detected later in cervical lymph nodes in various animal species.^44^ Therefore brain fluid effluent must exit the cranium and at least some of it must go via lymphatics if it reaches the lymph nodes to which the head drains. However, since the draining interstitial fluid was often assumed to mix with the CSF, the focus of most previous studies exploring the role of lymphatics in brain fluid drainage has largely been on CSF outflow from the cranium. Also, as noted previously, intraparenchymal administration is not feasible in humans which is why the few human experiments available have relied on intrathecal administration. There is strongest evidence that significant CSF outflow occurs through the cribriform plate, along dural membranes around basal cranial nerves as they exit the skull base, and via meningeal channels running along the major venous sinuses.^1^

An extensive network of lymphatic vessels has been identified along the dural meninges in animals and in vivo and at post mortem in humans.^4,37,45^ In experiments with transgenic mice lacking meningeal lymphatics and in mice where these had been ablated,^37,46^ there was impaired parenchymal clearance of macromolecules to cervical nodes. However, the functional significance of these lymphatic vessels is not fully elucidated and there might be differences in respect to location, e.g., dorsal, or basal.^45^ Rodent experiments using tracer delivered into the CSF or brain parenchyma suggest that fluid is preferentially drained by routes at the base of the brain rather than dorsal meningeal lymphatics.^37,47^ Also, morphological differences between the lymphatics of the basal and dorsal systems have been observed in mice, where the dorsal dural lymphatics lack valves, are smaller, often discontinuous, compared with basal brain lymphatics, all features that would presumably make the dorsal lymphatics less suitable for rapid high flow drainage.^47^

### Imaging study

#### Subject characteristics

The study included 19 subjects with mild ischaemic stroke at one to three months prior to MRI imaging. The median age was 62 years (IQR 49–68) and there were 13 males (68.4%). The majority of subjects had hypertension (59.9%) and/or hypercholesterolemia (84.2%), and 15.8% were diagnosed with diabetes mellitus. Most subjects had findings on MRI indicative of at least moderate SVD, including a median Fazekas score of 3 (IQR 1–5) and a median number of visible PVS in the centrum semiovale and basal ganglia of 4 (IQR 3–5).

#### Signal intensity before and after contrast in control areas

There was very little variation in SI in the ‘negative control’, NAWM, between individuals and little change on pre- versus post-contrast imaging: a median value of 188 (IQR 175*–*204) and 196 (IQR 176*–*211), respectively (p = 0.14). For the ‘positive control’, the pituitary stalk, there was a substantial increase in median SI, from 373 (IQR 338–410) pre-contrast to 618 (IQR 569–685) post-contrast (p < 0.001).

#### Signal intensity before and after contrast at areas of assessment

There was little variation in time from administration of contrast to image acquisition: between 20–30 minutes for all subjects except one (46 minutes), median 25 (IQR 23–27). This time was not significantly associated to the relative change in SI between pre- and post-contrast imaging (Std. Beta −0.29, p = 0.24).

**Figure 2.**
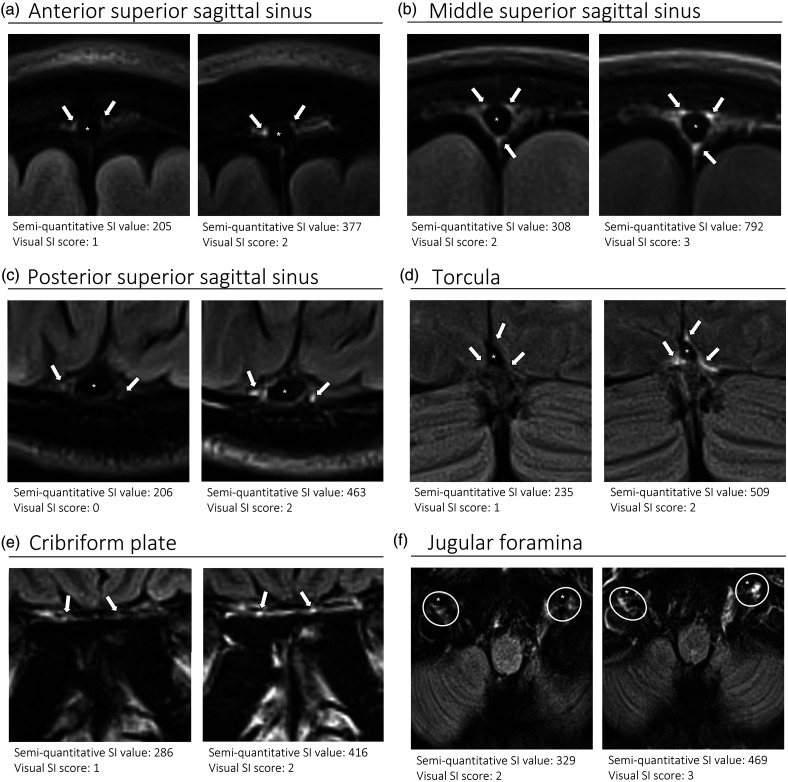
Representative T2 FLAIR images showing signal intensity in dorsal and basal structures. Pre- (left side of all panels) and post-contrast (right side of all panels) in the same subject for each area of assessment (panels a–f) (different subjects for different areas).The measured semi-quantitative SI values in arbitrary units (the average of all regions of interest measured at the specific area) and qualitative, visual SI scores are indicated below the images from the individual subjects. For the areas along the superior sagittal sinus (SSS) there were punctate hyperintensities at either side and inferiorly to the sinus, representing meningeal lymphatics in cross section. The hyperintensities at the cribriform plate and jugular foramina were more diffuse. For the latter, the white circles indicate the area of the pars nervosa and not individual regions of interest. The arrows and asterisks indicate meningeal lymphatic structures and the lumen of venous vessels (SSS, jugular vein), respectively. SI: signal intensity.

At all areas of assessment (along the SSS, superficial cortical perivenous spaces, at the cribriform plate, and at the jugular foramina) there were structures discernible from the surrounding CSF/tissue on pre-contrast imaging suspected to be involved in fluid outflow (Figure 2). These displayed distinct increases in SI on FLAIR 20–30 minutes after the administration of contrast. The hyperintensities along the SSS, assessed in cross section, showed a characteristic pattern, most often with one punctate hyperintensity to either side of the sinus, and sometimes one inferiorly, forming a triangular pattern (Figure 2(a) to (c)). The hyperintensities at the cribriform plate were also punctate in cross-section but more irregular in distribution, whereas the hyperintensities at the jugular foramina were more diffuse (Figure 2(e) and (f)).

**Figure 3.**
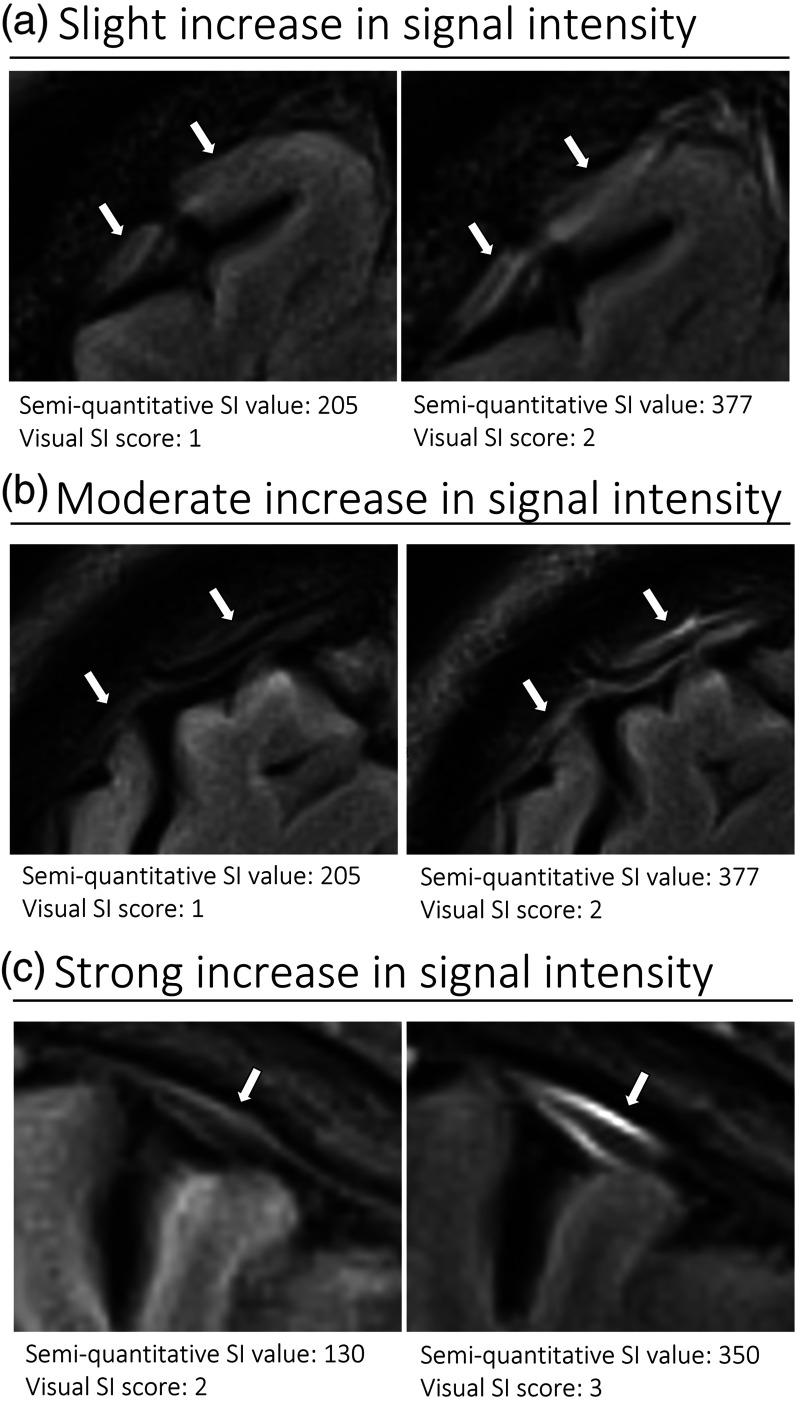
Representative T2 FLAIR images of superficial cortical perivenous spaces. Pre- (left side of all panels) and post-contrast (right side of all panels) images in the same subject showing slight (a), moderate (b), and strong (c) increase in SI (different subjects for a, b, and c). The measured semi-quantitative SI values in arbitrary units (the average of all regions of interest measured at the specific area) and qualitative, visual SI scores are indicated below the images. The arrows indicate the perivenous spaces. SI: signal intensity.

The number of hyperintense superficial cortical perivenous spaces, assessed in longitudinal section (Figure 3(a) to (c)) was highly variable between individuals. In total, 44 vessels were included from 16 subjects (in three subjects the visible perivenous spaces were shorter than two cm, therefore not fulfilling the inclusion criteria), with a median number of two per individual (IQR 1–3, range 0–7). The distribution of all perivenous spaces meeting the criteria was evenly split between the right and left hemisphere (26 and 18, respectively) as well as between the frontal, parietal, and occipital regions (13, 13 and 18, respectively).

**Figure 4.**
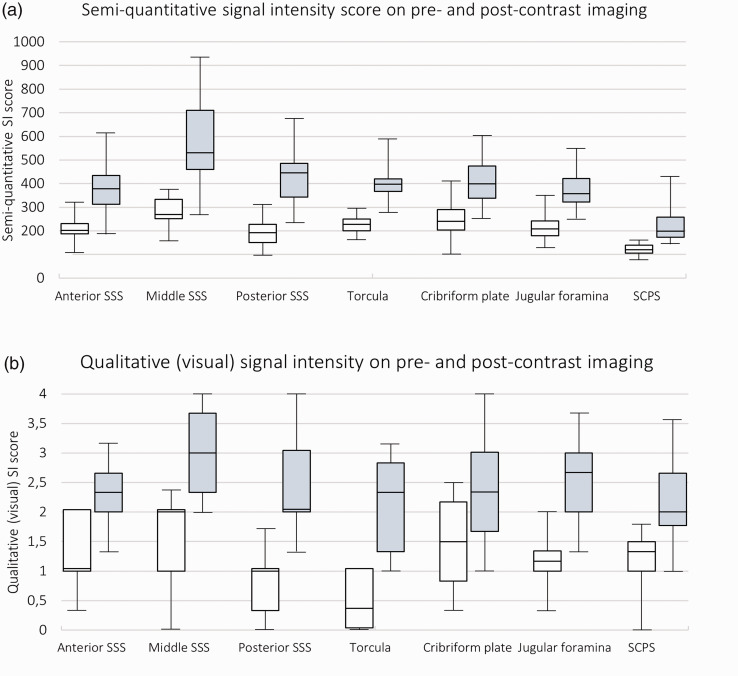
Box plot diagrams showing signal intensity in the different areas of assessment. Pre- (white) and post-contrast (grey) semi-quantitative SI values in arbitrary units (a) and qualitative (visual) SI score (b). Median (25th–75th percentile). N = 19 except for analyses on perivenous spaces where there were three patients missing. SCPS: superficial cortical perivenous spaces; SI: signal intensity; SSS: superior sagittal sinus.

For all areas of assessment, the increase in SI from pre- to post-contrast imaging, measured semi-quantitatively was highly significant (p < 0.001) and greatest for the dorsal areas. See Figure 4(a) for a box plot diagram and Table 2 for exact values. The median increase in SI in relation to pre-contrast SI was 94% for the Torcula, 82% for the anterior SSS, 104% for the middle SSS, 119% for the posterior SSS, and 96% for the superficial cortical perivenous spaces whereas it was lower in the basal areas: the cribriform plate (67%) and the jugular foramina (72%).

**Table 2.** Median signal intensity (pre- and post-contrast) in arbitrary units as well as absolute and relative change.

	Superior sagittal sinus			Torcula	Cribriform plate	Jugular foramen	Perivenous spaces
	Anterior	Middle	Posterior				
Signal intensity							
Pre-contrast, median (IQR)	202 (188–231)	270 (252–334)	193 (151–228)	227 (200–249)	240 (203–291)	209 (180–242)	120 (106–140)
Post-contrast, median (IQR)	379 (313–434)	532 (463–714)	449 (346–489)	427 (386–449)	400 (338–474)	357 (324–421)	209 (173–269)
Absolute change, median (IQR)	155 (125–197)	272 (202–364)	244 (158–280)	191 (150–256)	156 (66–200)	161 (111–177)	95 (52–150)
Change relative to pre-contrast signal intensity, median (IQR)	82% (67–101)	104% (75–117)	119% (112–142)	94% (67–115)	67% (25–86)	72% (48–89)	96% (41–131)

Average of the three measurements.

IQR: interquartile range.

Also, post-contrast SI in the Torcula, anterior SSS, middle SSS, and posterior SSS were significantly correlated to each other, whereas no such correlation was found between other areas of assessment (Supplementary table 3). However, there were no significant correlation between relative change in SI in different areas of assessment (except between the Torcula and the middle SSS).

The qualitative, visual scoring of SI followed a similar pattern, with highly significant increases between pre- and post-contrast imaging for all areas of assessment (p < 0.001) (See Figure 4(b) for a box plot diagram and supplementary table 5 for exact values). Plotting the average SI measured semi-quantitatively against the qualitative, visual scoring for each area of assessment showed a strong association (Std. Beta = 0.80, p < 0.001).

#### Signal increase in relation to patient characteristics and SVD features

**Figure 5.**
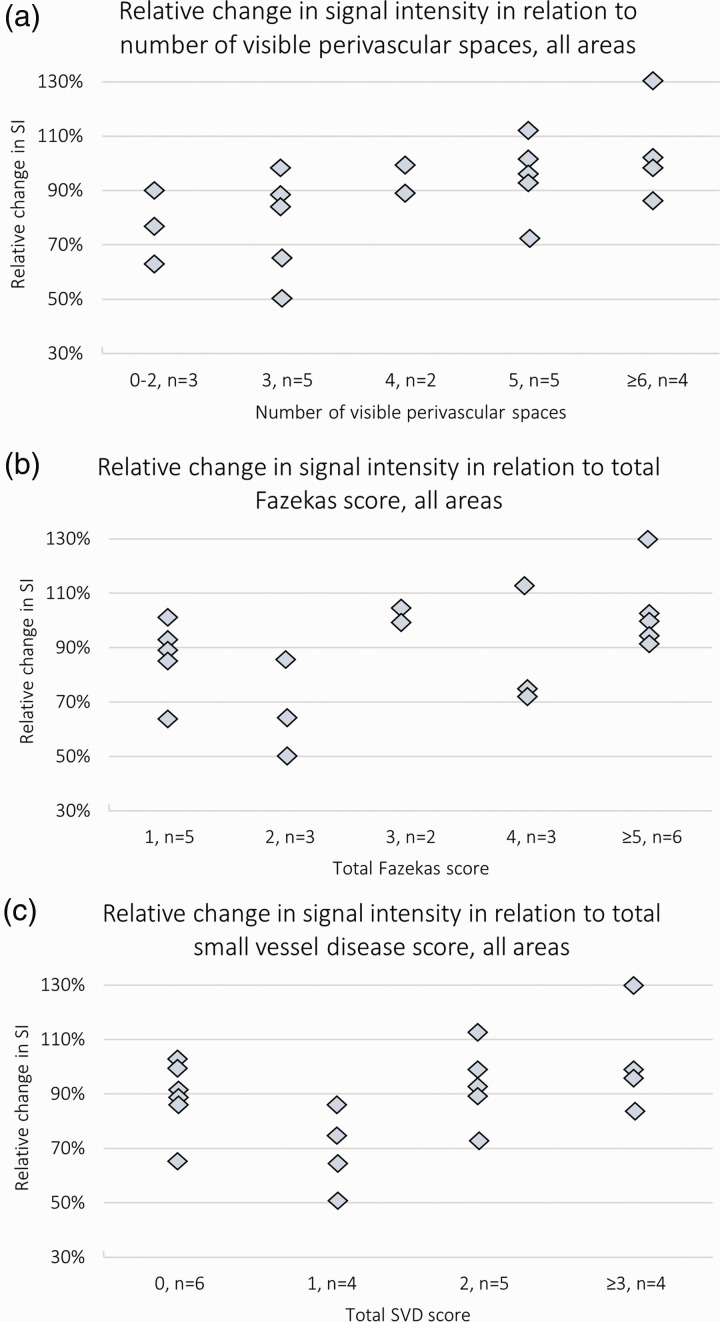
Scatter plot diagrams showing the change in semi-quantitative signal intensity relative to markers of small vessel disease in each subject. The average change from all seven sites of measurement (as a proportion of the pre-contrast SI) in relation to the total number of visible perivascular spaces in the centrum semiovale and the basal ganglia (a), the total Fazekas score (b), and the total small vessel disease score (c). N = 19. SI: signal intensity; SVD: small vessel disease.

To assess the association between SVD and relative change in semi-quantitative SI values (the average for all seven areas of measurement), we performed separate linear regression analyses for PVS, Fazekas score and total SVD score, adjusting for age and sex. There was a significant positive association of SI increase with the number of visible PVS in the centrum semiovale and basal ganglia (Std. Beta 0.71, p = 0.01, Figure 5(a)), no definite association with the Fazekas score (Std. Beta 0.37, p = 0.18, Figure 5(b)), and a borderline significant positive association with the total SVD score (Std. Beta 0.48, p = 0.08, Figure 5(c)). In a separate model only including age and sex, neither age nor sex showed a significant association to relative change in SI (Std. Beta 0.14, p = 0.58 and Std. Beta 0.01, p = 0.95, respectively).

#### Reliability measures

All measurements of SI were repeated three times. For the semi-quantitative measurement, the mean difference between each of the three pairs of values in proportion to the absolute SI value was under 10% for all areas of measurement, except for Torcula post-contrast (0.4–12.7%), the cribriform plate post-contrast (0.5–18%) and jugular foramen pre-contrast (0.4–22.3%) (Supplementary table 4). (There was significant variation between the first and the other two (second/third) scorings for the Torcula post-contrast, the jugular foramen pre-contrast and cortical superficial perivenous spaces post-contrast. As assessed by the ICC there was good reliability (a coefficient greater than 0,75) pre- and post-contrast for all areas of assessment except for the jugular foramen where there were values of around 0.7 for both pre- and post-contrast, indicating moderate reliability. The discrepancy between values did not vary significantly through the range of measurements. For the qualitative, visual scoring, there was greater reliability for the dorsal areas (mostly moderate values: Fleiss’ kappa >0.4), than for the basal areas (cribriform plate and the jugular foramen) and the superficial cortical perivenous spaces where the reliability was poor (Fleiss’ kappa < 0.4) (Supplementary table 5).

## Discussion

Following IV administration of Gd contrast, at 20–30 minutes, the SI was greatly increased (p < 0.001) in cortical superficial perivenous spaces, along the SSS (a finding generally accepted to represent dorsal meningeal lymphatics^4,16^) and at basal areas (cribriform plate and jugular foramina). The increase was largest for the dorsal areas along the SSS, which also showed significant correlation with each other. The repeatability of the measures was good, and we found potential associations of SI with SVD features in this small pilot study.

However, the pathway of the intravenous contrast from the peripheral circulation to these areas is not clear and may or may not involve the brain parenchyma. Contrast might enter the brain interstitial space directly from the vasculature by crossing the BBB, or it might first pass into the CSF from the choroid plexus during formation of CSF and thence flow into the brain parenchyma via periarteriolar spaces. In principle, the BBB is not supposed to allow Gd to cross. However, the BBB becomes subtly leaky with advancing age,^48^ and in a range of pathological conditions,^49^ including a diffuse leakage which can be appreciated with advanced imaging techniques in patients with SVD.^50^ However, there would have to be a major concentrating effect of contrast since there was very little enhancement in the NAWM. The contrast would then be drained from the brain parenchyma to the dorsal meningeal lymphatics, possible via perivenous spaces, which showed significant enhancement in our study. Hyperintensities around superficial cortical veins have previously been reported, e.g., by Naganawa et al.^9^ which they interpreted as possible leakage from the superficial cortical vein walls, which might be a possible explanation for the findings in our study. But while the authors suggested the leakage to occur into the surrounding tissue our images show that the contrast agent is mostly contained in a perivenous compartment separate from the CSF with a ‘tramline’ appearance and does not appear to spread out more widely. Meningeal lymphatics could then fill from the perivenous spaces (our human MRI is not detailed enough to resolve this clearly in all cases) as described by Naganawa et al.^51^ Thus, the fluid might leak directly to the perivenous spaces and then drain to the meningeal vessels, bypassing the brain parenchyma.

However, there are several alternative explanations for the enhancement of dural meningeal lymphatics not involving the brain parenchyma or the perivenous spaces. For instance, there might be passage of contrast to the dural interstitium directly from the CSF. However, the presence of the arachnoid barrier separating the CSF from the dural interstitium speaks against that this pathway would contribute to significant fluid passage. Alternatively, there might be movement of contrast into the dural interstitium directly from dural capillaries.^4^ However, since the dura is a relatively avascular structure, particularly compared to the highly vascular brain, it may be unlikely that enough contrast would pass through dural arteries and dura alone to significantly raise the signal. These routes would not solve the question of what happens to fluid draining out of the brain.

As for fluid drainage via the basal routes (cribriform plate and jugular foramina), this seems to be quantitatively important, as suggested by previous evidence,^1^ although the focus has mostly been on CSF drainage. In our study the change in SI at these areas was smaller than at the dorsal areas and the enhancement pattern was less uniform and more diffuse as compared to the clear punctate pattern observed around the SSS. The enhancement in these basal areas might represent contrast in a range of different compartments: in fluid spaces, connective tissue, or lymphatics associated with cranial nerves or vessels,^1^ and the pathways moving fluid here are unclear.

Notably, despite the small sample size, we were able to detect associations between change in SI and markers of SVD. It is unclear what these associations represent. There are several possibilities, e.g.: (1) an increased flow of contrast and fluid into the parenchyma, possibly through a leaky BBB, known to be associated with increased PVS visibility and SVD severity and hence increased outflow, or (2) delayed drainage and stagnation of fluid and contrast. Interestingly, the association was particularly strong for the number of visible intraparenchymal PVS, already known to be involved in brain fluid management and BBB leakage.

In summary, the exact route of contrast from the peripheral circulation is unclear but our finding suggests that interstitial fluid drains from brain parenchyma to meningeal lymphatic vessels along the SSS, possibly via superficial cortical perivenous spaces, kept separate from the CSF spaces.

### Limitations

The number of subjects was small: only 19 individuals. Thus, true associations might not have been detected, e.g., between markers of SVD and change in SI. Also, measurement of SI in the basal outflow sites (the cribriform plate and in particular the jugular foramina) showed less favourable reliability characteristics, indicating that our results from these areas might be less accurate. However, we acknowledge that there does not exist a single best method of assessing reliability for our study which is why we chose to include several methods. The varying characteristics of the methods is illustrated by a slight discrepancy in results. Also, the proposed cut-offs for level of reliability for the ICC and Fleiss’ kappa are debated and should only be viewed as a rough indication. Further, our measurement of SI is only to be considered semi-quantitative and no normalisation was performed. However, there was very little variation in SI in our ‘negative control’, NAWM, between individuals and little change on pre- versus post-contrast imaging, whereas the ‘positive control’ reference tissue (the pituitary stalk) showed strong signal increase after contrast. Nevertheless, we focus on group differences and relative change in SI with each patient effectively acting as their own control. Comparisons of absolute values on an individual level cannot reliably be made with this method.

### Future research

Even though brain fluid drainage has been studied for over a century, knowledge is still rudimentary and experimentation in humans limited by invasiveness. However, the increasing range and sophistication of available imaging techniques have the potential to advance understanding. This study successfully demonstrates the feasibility of quantifying brain fluid outflow pathways in humans using very accessible techniques. A similar approach to assessment of the meningeal lymphatics along the sagittal sinus was recently published by another group in normal volunteers, finding similar SI change post-contrast imaging.^15^ We extend this approach by also assessing other well described and potentially visible drainage points. However, in both studies, the sample was small which limits in-depth analysis. Future research should try to use a similar methodology in a larger sample of subjects, perhaps including groups with neurodegenerative conditions such as Alzheimer’s or Parkinson’s disease, as well as increased pressure states such as normal pressure hydrocephalus or idiopathic intracranial hypertension. Also, several additional important aspects need to be addressed, such as the optimal timing for image acquisition in relation to contrast administration, the signal change in different anatomical locations from early through longer times after injection, and the relationship between the various areas of drainage and different anatomical brain regions. Importantly, reflecting the more diffuse distribution of the post-contrast hyperintensities at the basal areas, there were slightly less favourable reliability characteristics. Together with a less clear anatomical and functional understanding, this might suggest that future research should try to improve on image acquisition and analysis of these difficult structures or focus on the dorsal areas. Our results show that post-contrast SI in the Torcula, anterior SSS, middle SSS, and posterior SSS were significantly correlated to each other, which suggests that measurement might be limited to fewer areas. Also, we did not assign different weights to specific areas since the relative physiological importance of each is currently poorly defined. This remains to be determined in larger studies. Further, we did not measure perivenous SI where the length of the vein was less than two cm; however, it should be possible to measure perivenous SI in all cases by relaxing this rule while maintaining good reliability in future studies. Finally, in our study, measurement was done manually and thus was very time consuming, but it might be possible to develop automatic measurement, particularly of the SI of the dorsal meningeal lymphatics which appear to be more uniform in morphology and distribution across individuals.

## Supplemental Material

sj-pdf-1-jcb-10.1177_0271678X231179555 - Supplemental material for Visualising and semi-quantitatively measuring brain fluid pathways, including meningeal lymphatics, in humans using widely available MRI techniques Click here for additional data file. Supplemental material, sj-pdf-1-jcb-10.1177_0271678X231179555 for Visualising and semi-quantitatively measuring brain fluid pathways, including meningeal lymphatics, in humans using widely available MRI techniques by Stefan Sennfält, Michael J Thrippleton, Michael Stringer, Carmen Arteaga Reyes, Francesca Chappell, Fergus Doubal, Daniela J Garcia, Junfang Zhang, Yajun Cheng and Joanna Wardlaw in Journal of Cerebral Blood Flow & Metabolism

## Data Availability

The data presented in this paper can be requested from the authors after attainment of the appropriate ethics approval.
