# Supplementary material for: Visualising and semi-quantitatively measuring brain fluid pathways, including meningeal lymphatics, in humans using widely available MRI techniques
Source: J Cereb Blood Flow Metab. 2023 May 31;43(10):1779–95. doi: 10.1177/0271678X231179555 (PMC10581238; doi:10.1177/0271678X231179555)
Supplement: sj-pdf-1-jcb-10.1177_0271678X231179555 - Supplemental material for Visualising and semi-quantitatively measuring brain fluid pathways, including meningeal lymphatics, in humans using widely available MRI techniques [file sj-pdf-1-jcb-10.1177_0271678X231179555.pdf]

Supplementary material

Visualising and semi-quantitatively measuring brain fluid pathways, including meningeal lymphatics, in humans using widely available MRI techniques

Supplementary table 1. Overview of the initial search process.

Date	Search terms	Filters	Resulting number of papers	Number deemed as relevant and assessed in detail
2022-05-30	Glymphatic system	Only review papers, Last five years	261	10
2022-06-06	Brain fluid homeostasis	Only review papers, Last five years	210	14
2022-06-06	Brain fluid transport	Only review papers, Last five years	290	15
2022-06-12	Meningeal lymphatics	Only review papers, Last five years	107	20
2022-06-12	Meningeal lymphatics imaging	Only review papers, Last five years	19	3
2022-06-15	Glymphatic imaging	Only review papers, Last five years	78	3

Supplementary table 2. Example images guiding qualitative (visual) scoring (1–4) of signal intensity in the different areas of assessment.

	Signal intensity score			
	1	2	3	4
Superior Sagittal Sinus (Middle)	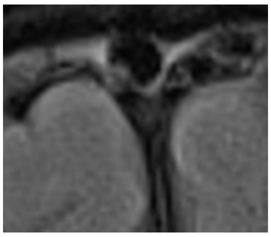	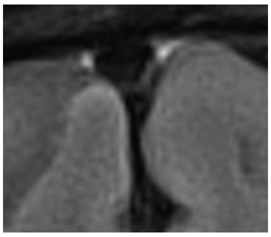	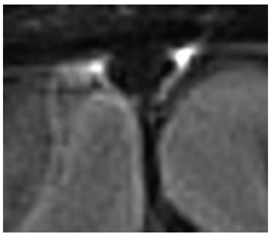	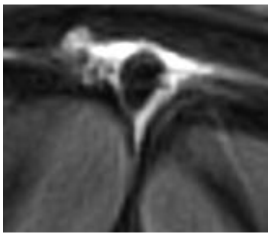
Torcula	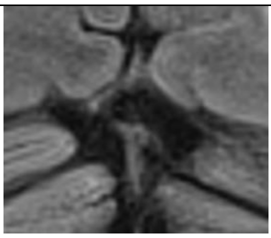	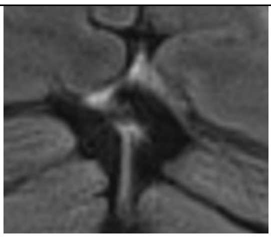	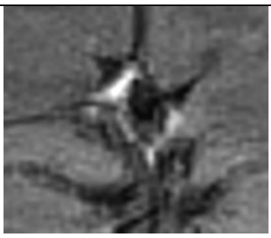	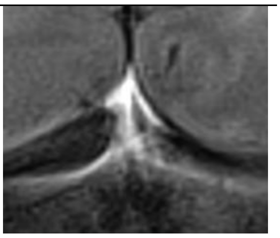
Cribriform plate	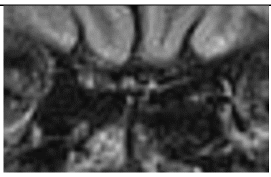	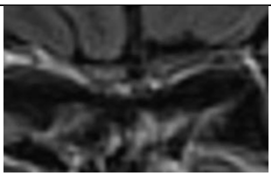	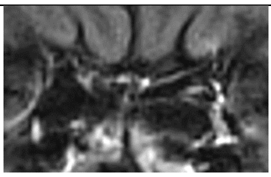	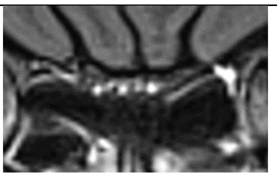
Jugular foramen	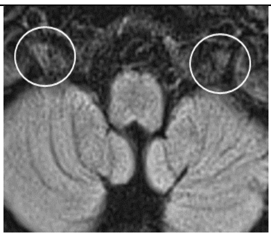	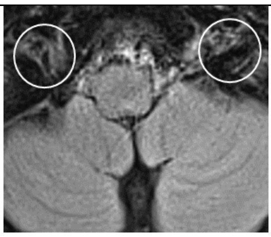	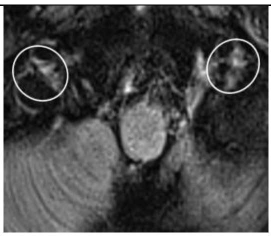	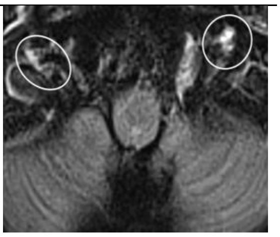
Superficial cortical perivenous spaces	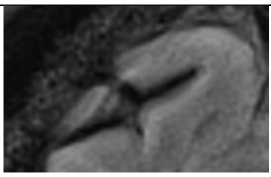	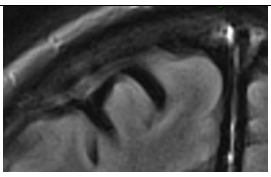	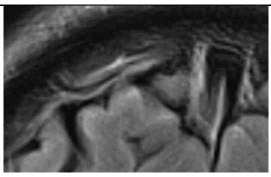	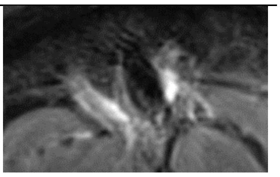

Supplementary table 3. Correlation matrix of absolute post-contrast semi-quantitative signal intensity (SI) and in change in SI between pre- and post-contrast measurement (relative to pre-contrast absolute SI) in areas of assessment. Pearson's correlation coefficient. N=19. There was significant correlation between the dorsal areas (the Torcula and the anterior, middle, and posterior superior sagittal sinus).
SI=Signal intensity.

	Superior sagittal sinus			Torcula	Cribriform plate	Jugular foramen	Superficial cortical perivenous spaces
	Anterior	Middle	Posterior				
**Superior sagittal sinus**							
**Anterior**							
Post-contrast SI	1	**0.54***	**0.67****	**0.49***	0.05	0.33	0.20
Change in SI	1	0.07	0.17	-0.05	0.01	0.05	0.02
**Middle**							
Post-contrast SI	**0.54***	1	**0.65****	0.41	0.07	**0.58***	0.10
Change in SI	0.07	1	0.34	**0.56***	0.03	-0.09	-0.08
**Posterior**							
Post-contrast SI	**0.67****	**0.65****	1	0.33	-0.08	0.09	0.05
Change in SI	0.17	0.34	1	0.38	0.40	-0.17	0.12
**Torcula**							
Post-contrast SI	**0.49***	0.41	0.33	1	0.03	0.24	0.37
Change in SI	-0.05	**0.56***	0.38	1	0.09	0.07	0.34
**Cribriform plate**							
Post-contrast SI	0.05	0.07	-0.08	0.03	1	0.10	-0.10
Change in SI	0.01	0.03	0.40	0.09	1	-0.07	-0.19
**Jugular foramen**							
Post-contrast SI	0.33	**0.58***	0.09	0.24	0.11	1	0.27
Change in SI	-0.06	-0.14	-0.31	-0.05	0.06	1	0.01
**Superficial cortical perivenous spaces ^^**							
Post-contrast SI	0.20	0.10	0.05	0.37	-0.10	0.27	1
Change in SI	0.02	-0.08	0.12	0.34	-0.19	0.01	1

*p<0.05, **p<0.01, ^^n=16

Supplementary table 4. Median semi-quantitative signal intensity values in arbitrary units (pre- and post-contrast with the interquartile range [IQR]) on the first, second, third measurement and reliability measures: mean differences between each pair of measurements and intraclass correlation coefficient [ICC] of all three. N=19.
CI=confidence interval, ICC= intraclass correlation coefficient, IQR=interquartile range

	Measurement				Mean differences (95% CI) between pairs of measurements. Absolute numbers *Proportional to the mean of the two measurements*			ICC
	1st	2nd	3rd	Mean	1st - 2nd	1st - 3rd	2nd–3rd	
Superior sagittal sinus								
Anterior								
Pre-contrast	205 (181–275)	205 (175–254)	197 (171–229)	202 (188–231)	-6.8 (-31.0–17.4)<br>*-3.1 (14.4–7.9)*	9.8 (-6.7–26.2)<br>*4.7 (-3.2–12.5)*	16.5 (-3.1–36.2)<br>*7.8 (-1.5–17.1)*	0.88
Post-contrast	401 (334–439)	378 (289–439)	368 (316–393)	379 (313–434)	-8.1 (-32.1–15.8)<br>*-4.2 (-16.5–8.2)*	13.0 (-11.7–37.6)<br>*7.1 (6.0–20.1)*	21.1 (0.1–42.1)<br>*9.8 (0.1–22.3)*	0.95
Middle								
Pre-contrast	295 (242–324)	273 (239–330)	269 (229–320)	270 (252–334)	0.7 (-19.0–20.5)<br>*0.5 (-13.3–14.0)*	12.2 (-6.3–30.6)<br>*8.9 (-0.5–21.9)*	11.4 (-9.6–32.5)<br>*8.3 (-7.0–24.6)*	0.91
Post-contrast	544 (483–693)	521 (428–665)	513 (458–639)	532 (463–714)	40.8 (-4.9–86.5)<br>*7.1 (-0.9–15.1)*	35.7 (3.0–68.5) *<br>*6.2 (0.5–11.9) **	-5.1 (-42.4–32.2)<br>*-0.9 (-7.6–5.8)*	0.96
Posterior								
Pre-contrast	192 (155–226)	188 (158–252)	193 (162–227)	193 (151–228)	-1.0 (-14.2–12.2)<br>*-0.5 (-7.2–6.2)*	-6.3 (-22.3–9.8)<br>*-3.2 (-11.0–5.0)*	-5.3 (-20.4–9.8)<br>*-2.6 (-10.1–4.9)*	0.96
Post-contrast	463 (358–499)	462 (349–499)	432 (325–469)	449 (346–489)	3.6 (-16.8–24.1)<br>*0.8 (-3.8–5.5)*	26.8 (-2.4–56.0)<br>*6.2 (-0.6–13.1)*	23.2 (-4.3–50.8)<br>*5.4 (-1.0–11.7)*	0.96
Torcula								
Pre-contrast	231 (221–265)	240 (194–266)	219 (194–238)	227 (200–249)	1.8 (-16.1–19.7) *<br>*0.8 (-6.9–8.2)*	17.0 (1.7–32.4)<br>*7.6 (0.8–14.5)*	15.3 (-4.0–34.5)<br>*6.8 (-1.8–15.3)*	0.80
Post-contrast	433 (403–452)	404 (368–442)	430 (389–481)	427 (386–449)	34.9 (10.1–59.7) **<br>*8.2 (2.3–14.0) ***	-1.6 (-22.7–19.5) **<br>*-0.4 (-5.2–4.3) ***	-36.5 (-59.5–13.5)<br>*-12.7 (-21.1–4.8)*	0.92
Cribriform plate								
Pre-contrast	278 (218–303)	235 (186–300)	244 (209–303)	240 (203–291)	12.6 (-20.7–46.0)<br>*0.5 (-7.9–18.3)*	9.6 (-15.2–34.4)<br>*0.4 (-0.6–13.4)*	-3.1 (-33.7–27.6)<br>*-0.1 (-13.3–10.9)*	0.90
Post-contrast	380 (304–479)	375 (302–486)	427 (329–455)	400 (338–474)	-17.9 (-65.8–30.0)<br>*-0.5 (-18.0–8.3)*	-32.4 (-81.0–16.2)<br>*-8.5 (21.4–4.2)*	-68.7 (-142.7–5.3)<br>*-18.0 (-37.6–0.1)*	0.86

**Jugular foramen**								
Pre-contrast	242 (179–273)	204 (177–235)	179 (156–242)	209 (180–242)	40.6 (3.8–77.3) *<br>*18.2 (0.2–0.35) **	47.5 (17.8–77.3) **<br>*22.3 (0.9–36.7) ***	6.9 (-20.9–34.8)<br>*0.4 (-11.1–18.4)*	0.71
Post-contrast	353 (296–469)	343 (260–385)	372 (315–437)	357 (324–421)	39.3 (-8.7–87.4)<br>*10.0 (-0.2–24.0)*	2.7 (-47.8–53.3)<br>*0.1 (12.2–13.8)*	-36.6 (-77.0–3.7)<br>*-9.8. (-21.3–0.1)*	0.73
**Superficial cortical perivenous spaces ^^**								
Pre-contrast	114 (101–138)	120 (102–146)	120 (103–147)	120 (33)	-7.0 (-14.1–0.2)<br>*-0.6 (-11.5–0.2)*	-7.2 (-24.9–10.6)<br>*-0.6 (-20.5–8.2)*	-0.2 (-14.1–13.8)<br>*-0.2 (-11.2–11.0)*	0.82
Post-contrast	183 (163–261)	223 (180–287)	221 (178–273)	209 (95)	-23.2 (-35.2—11.2) **<br>*-10.0 (-15.4–0.5) ***	-22.3 (-35.2—9.5) **<br>*-9.7 (-15.4–0.4) ***	0.9 (-10.5–12.3)<br>*0.1 (-0.4–0.5)*	0.98
**Normal-appearing white matter**								
Pre-contrast	183 (179–199)	190 (176–204)	188 (169–207)	188 (175–204)	14.1 (-14.3–42.4)<br>*7.5 (-7.5–22.6)*	24.1 (-7.4–55.5)<br>*13.0 (-4.0–30.0)*	10.0 (-21.0–41.0)<br>*5.3 (-11.1–21.7)*	0.99
Post-contrast	200 (175–215)	193 (179–213)	191 (178–206)	196 (176–211)	-2.1 (-7.1–2.8)<br>*-0.1 (-3.6–1.4)*	-0.9 (-5.4–3.6)<br>*-0.4 (-2.8–1.9)*	1.2 (-2.1–4.5)<br>*0.6 (1.1–2.3)*	0.98
**Pituitary stalk**								
Pre-contrast	378 (335–461)	386 (342–425)	351 (324–426)	373 (338–410)	14.3 (-14.3–42.4)<br>*3.7 (-3.7–11.4)*	24.1 (-7.4–55.5)<br>*6.7 (-2.1–15.4)*	10.0 (-21.0–41.0)<br>*2.8 (-5.8–11.4)*	0.81
Post-contrast	625 (590–689)	654 (570–693)	622 (529–659)	618 (569–685)	23.3 (-13.0–59.5)<br>3.6 (-2.0–9.3)	35.9 (-8.1–79.8)<br>*5.8 (-1.3–12.9)*	12.6 (-39.7–64.9)<br>*2.0 (-6.1–10.2)*	0.78

* Significant deviation from zero p<0.05, ** significant deviation from zero p< 0.01, ^^ n=16

Supplementary table 5. Median qualitative (visual) signal intensity scores (pre- and post-contrast) on the first, second, and third measurement and reliability measures (Fleiss' kappa) at the different areas of assessment. Reported as mean (95% confidence interval). N=19

	1st scoring	2nd scoring	3rd scoring	Mean	Fleiss' kappa
Superior sagittal sinus					
Anterior					
Pre-contrast	1.47 (1.23–1.72)	1.26 (0.99–1.53)	1.26 (0.99–1.15)	1.33 (1.09–1.57)	0.65
Post-contrast	2.47 (2.18–2.80)	2.37 (2.08–2.66)	2.26 (1.95–2.58)	2.37 (2.10–2.64)	0.37
Middle					
Pre-contrast	1.63 (1.34–1.92)	1.63 (1.30–1.96)	1.63 (1.34–1.92)	1.63 (1.34–1.92)	0.79
Post-contrast	3.16 (2.83–3.49)	3.00 (2.57–3.43)	3.00 (2.64–3.36)	3.06 (2.72–3.39)	0.62
Posterior					
Pre-contrast	0.89 (0.67–1.12)	0.63 (0.34–0.92)	0.74 (0.42–1.05)	0.75 (0.53–0.98)	0.32
Post-contrast	2.47 (2.07–2.88)	2.37 (1.97–2.77)	2.32 (1.96–2.68)	2.39 (2.04–2.73)	0.46
Torcula					
Pre-contrast	0.74 (0.52–0.95)	0.42 (0.18–0.67)	0.37 (0.13–0.61)	0.51 (0.31–0.70)	0.44
Post-contrast	2.37 (2.04–2.70)	1.84 (1.47–2.21)	1.95 (1.57–2.32)	2.05 (1.73–2.38)	0.36
Cribriform plate					
Pre-contrast	1.37 (1.00–1.74)	1.21 (0.87–1.55)	1.21 (0.83–1.59)	1.26 (0.94–1.58)	0.39
Post-contrast	2.16 (1.67–2.65)	2.32 (1.83–2.80)	2.16 (1.68–2.65)	2.28 (1.83–2.73)	0.30
Jugular foramen					
Pre-contrast	1.29 (1.02–1.56)	1.26 (0.99–1.53)	1.16 (0.87–1.45)	1.24 (1.02–1.45)	0.25
Post-contrast	2.34 (2.00–2.73)	2.47 (2.04–2.91)	2.58 (2.18–2.98)	2.46 (2.16–2.78)	0.25
Superficial cortical perivenous spaces ^^					
Pre-contrast	1.11 (0.88–1.34)	1.28 (1.01–1.55)	1.24 (0.98–1.50)	1.23 (0.99–1.47)	0.32
Post-contrast	2.05 (1.67–2.42)	2.30 (1.98–2.63)	2.90 (1.96–2.62)	2.20 (1.84–2.55)	0.9

^^ n=16

List of members of the Mild Stroke Study 3 (MSS-3) Investigator group

Una Clancy[1]
Daniela Jaime Garcia[1]
Carmen Arteaga[1]
Michael Stringer[1]
Michael J. Thrippleton[1]
Maria C. Valdés-Hernández [1]
Stewart Wiseman[1]
Susana Muñoz Maniega[1]
Olivia K.L. Hamilton[1]
Francesca M. Chappell[1]
Rosalind Brown[1]
Gordon Blair[1]
Will Hewins[1]
Emilie Sleight[1]
Tom MacGillivray[1]
Charlene Hamid[1]
Alasdair G. Morgan[1]
Cameron Manning[1]
Angela Jochems[1]
Ellen Backhouse[1]
Dillys Liu, Hong Kong, China
Junfang Zhang, Shanghai, China
Yajun Cheng, Chengdu, China
Iona Hamilton[1]
Dominic Job[1]
David Perry[1]
Ian Marshall[1]
Rachel Penman[1]
Fergus N. Doubal[1]
Joanna M. Wardlaw[1]

[1] Centre for Clinical Brain Sciences, University of Edinburgh
